# “Look at You Having Fun With Your Markers in Here!”: Child Life Specialists’ Countering of Infantilizating Narratives in Adult Oncology

**DOI:** 10.1177/10497323241257399

**Published:** 2024-08-07

**Authors:** Monica L. Molinaro, Shipra Taneja, David L. Lysecki, Heather McKean, Daryl Bainbridge, Jonathan Sussman, Meredith Vanstone

**Affiliations:** 1 Institute of Health Sciences Education, 5620 McGill University, Montreal, QC, Canada; 2 Department of Family Medicine, 3710 McMaster University, Hamilton, ON, Canada; 3 Department of Pediatrics, 3710 McMaster University, Hamilton, ON, Canada; 4 Hamilton Health Sciences, 103398 McMaster Children’s Hospital, Hamilton, ON, Canada; 5 Department of Oncology, 3710 McMaster University, Hamilton, ON, Canada

**Keywords:** oncology, cancer, child life, child life specialist, narrative, counter-story

## Abstract

Child life specialists are clinically trained and educated healthcare professionals who work in both healthcare environments and the community to address the needs of ill children and their families. However, child life specialists have previously reported potential for their role, responsibilities, and scope of practice to be misunderstood by their clinical colleagues. Using a narrative methodology, this paper presents the composite narrative of Diane, whose story encompasses the stories of the four child life specialists working in adult oncology environments in Ontario, Canada. Diane’s narrative is a counter-story, which counters common assumptions, beliefs, and attitudes about child life specialists. Through spending significant time narrating the multitude of tasks that are encompassed within her scope of care, Diane reaffirms her identity as a valuable member of an interprofessional adult oncology team and counters infantilizing assumptions that she is merely a babysitter or child entertainer. Her story highlights how, while the introduction of child life specialists to adult healthcare environments is new, the work they do is of great benefit to families and their children. The lack of understanding from clinical colleagues of the role of child life specialists, however, hinders not only the development of relationships between colleagues, but also the care for these families.

## Introduction

Prioritizing and improving the care of children in hospitals has been an endeavor taken on by healthcare professionals, researchers, and policymakers alike for over a century (Association of Child Life Professionals, 2023a; Ricks & Faubert, 1981). Within this time period, one of the significant developments in child healthcare was the inception of the child life profession, particularly the establishment of the child life specialist role in 1982 (Association of Child Life Professionals, 2023a, 2023b). The professional mission and vision and practice standards of child life specialists focus on family-centered care and developmentally appropriate interventions (Association of Child Life Professionals, 2020). These interventions strive to optimize the experience of hospital stays or medical procedures for children, given the circumstances and contexts of the child’s health issues (Association of Child Life Professionals, 2023a, 2023b; Bassin, 2022; Canadian Association of Child Life Leaders, 2022; McMaster University Masters in Child Life & Pediatric Psychosocial Care Program, 2023).

Child life specialists are clinically trained and educated healthcare professionals who work to address the needs of both ill children and their families and provide developmentally appropriate care (Association of Child Life Professionals, 2023a, 2023b; Bassin, 2022; Canadian Association of Child Life Leaders, 2022; McMaster University Masters in Child Life & Pediatric Psychosocial Care Program, 2023). Working in both healthcare and community settings, child life specialists have unique and specific post-secondary training (i.e., didactic and practical training through baccalaureate and/or master’s degrees, clinical internships under the supervision of another child life specialist, and a certification exam) that aims to reduce the negative impacts of hospital stays, medical procedures, and other forms of medical care for children and their families (Association of Child Life Professionals, 2023a, 2023b; Bassin, 2022; Canadian Association of Child Life Leaders, 2022; McMaster University Masters in Child Life & Pediatric Psychosocial Care Program, 2023). To do this, multiple interventions are used to help children develop coping, emotional regulation, and communication skills and make a hospital stay, injury, or medical procedures less stressful for an entire family (Association of Child Life Professionals, 2023a, 2023b; Bassin, 2022; Canadian Association of Child Life Leaders, 2022; McMaster University Masters in Child Life & Pediatric Psychosocial Care Program, 2023).

Recently, child life specialists have been increasingly integrated into various adult healthcare settings, as there are limited resources available for adult clinical teams to support the children of families facing illness (Bruce & McCue, 2018; Lysecki et al., 2021; Piazza et al., 2021; Sutter & Reid, 2012). In adult healthcare settings, the child life specialist can help both children and their family members cope with and comprehend what it means to have an ill parent. To engage in this type of work, child life specialists must have extensive knowledge of not only the appropriate interventions adapted for the various developmental stages of the children, but also of the clinical contexts in which these children (and their families) are embedded. In the context of adult oncology, child life specialists address the needs of the children and adolescents of adult cancer patients, using a family-centered care approach to collaborate with the adult patient, other family members, and the healthcare team to support the well-being of the child (Lookabaugh & Ballard, 2018). Once the oncology team refers the family to the child life specialist, the child life specialist can provide the family, and more specifically the child(ren), with developmentally appropriate information about the diagnosis, ongoing treatment, and, in some cases, prognoses and information regarding death and dying from terminal illness.

While child life specialists have specialized roles that require hundreds of hours of clinical training, previous research has drawn attention to a longstanding misconception of the role and valuable work that child life specialists do by their colleagues in both pediatric and adult healthcare environments (Cole et al., 2001; Crider & Pate, 2011; Gaynard, 1985; Holloway & Wallinga, 1990; Lookabaugh & Ballard, 2018; Munn et al., 1996; Wittenberg & Barnhart, 2021). In particular, child life specialists have previously reported being disregarded as members of the interprofessional healthcare team (Fehlen, 2016; Gaynard, 1985), going so far as to be perceived as babysitters or child entertainers among their clinical colleagues (Gaynard, 1985). These longstanding misunderstandings have been attributed to a lack of knowledge of the role and responsibilities of child life specialists across the rest of the interprofessional health team, including the mischaracterization that therapeutic play activities are merely forms of entertainment rather than clinical interventions to help with emotional regulation and communication (Cole et al., 2001; Crider & Pate, 2011; Gaynard, 1985; Holloway & Wallinga, 1990; Lookabaugh & Ballard, 2018; Munn et al., 1996; Wittenberg & Barnhart, 2021).

Much of the scholarly literature on the experiences of child life specialists have included descriptions of the value of their role, through the reporting of the effectiveness of particular interventions child life specialists have used (Crider & Pate, 2011; Drayton et al., 2019; LeBlanc et al., 2014) or through descriptions of child life specialist placement in differing clinical environments, such as an adult ICU (Crider & Pate, 2011) or pediatric emergency department (Krebel et al., 1996). However, there is a paucity of literature elucidating the importance of the role from the perspectives of child life specialists themselves, particularly those working in adult oncology environments.

One method of drawing attention to the importance of child life is through the presentation of child life specialists’ counter-stories. A counter-story, as originally conceptualized by Hilde Lindemann Nelson (1995, 2001), is “a story that resists an oppressive identity and attempts to replace it with one that commands respect” (Lindemann Nelson, 2001, p. 6, originally defined in Lindemann Nelson, 1995). Within the narratives of healthcare professionals, counter-stories have previously been used as acts of resistance by nurses to re-assign value and power to nursing care (Molinaro et al., 2023; Peter & Liaschenko, 2013). Similar to nursing, child life is a highly feminized profession (Zippia, 2022) that requires medical knowledge and expertise to provide relational and psychosocial care to children and their families (Association of Child Life Professionals, 2020, 2023a, 2023b; McMaster University Masters in Child Life & Pediatric Psychosocial Care Program, 2023). However, the work of child life specialists is often assigned less value by their clinical counterparts and institutions, reflecting societal perceptions of what constitutes “important” clinical work (Cole et al., 2001; Crider & Pate, 2011; Gaynard, 1985; Holloway & Wallinga, 1990; Lookabaugh & Ballard, 2018; Munn et al., 1996; Wittenberg & Barnhart, 2021). Child life specialists work in a healthcare system with firmly entrenched interprofessional hierarchies that value and resource particular roles over others through assignation of salary, clinical decision-making, and legitimate power to grant or revoke benefits to patients (Baker et al., 2011; Cohen Konrad et al., 2019; Lingard et al., 2012; Nugus et al., 2010; Paradis & Whitehead, 2015; Vanstone & Grierson, 2022).

In this article, we present a composite counter-story of four child life specialists working with children and families who have an adult family member with cancer in Ontario, Canada, that reaffirms their expertise and clinical training against infantilizing narratives and assumptions regarding their work. The purpose of our broader study was to explore the narratives of child life specialists working in adult oncology units (Taneja et al., 2023). We argue that the story presented in this paper functions as a counter-story through the lengthy narrations of skills, expertise, and active negotiations of identity that reaffirm child life specialists as skilled professionals who hold valuable clinical expertise for the adult oncology environment. This counter-story also draws attention to the frequent mischaracterization of child life specialists’ expertise by both families and their colleagues in an adult oncology environment. We then further describe how counter-stories were used in child life specialists’ daily work to educate families and colleagues on what it means to be, and the importance of being, a child life specialist.

## Methodology

Our chosen methodology to explore the stories of child life specialists was narrative inquiry, which encompasses a variety of approaches, methods, and assumptions that are all underlaid by an interest in stories (Riessman, 2008; Smith & Sparkes, 2006). In the context of qualitative research, narrative inquiry involves the collection of stories as a form of data for analysis (Emden, 1998), allowing for scrutiny of both the content and structure of a participant’s story. More information on our methodology and methods can be found in Taneja et al. (2023).

Narrative inquiry is underlaid by the assumption that storytelling acts as a way of constructing and negotiating our realities and selves (Mishler, 1986; Smith & Sparkes, 2006). One’s sense of self can be embedded within the ways that one narrates their connections to others (Bissell et al., 2006); when a person’s sense of identity is challenged or threatened, stories are a useful tool for making sense of the ambiguities and challenges that characterize the person’s relationships with others and with the structures within which they are embedded (Bruner, 1987; Emerson & Frosh, 2004; Riessman, 1993). In the case of the present study, narrative inquiry allowed for examination of the experiences of four child life specialists in an adult oncology unit and an examination of how their stories counter common assumptions about the value of the child life specialists and the knowledge, skills, and expertise involved in their work.

### Theoretical Lens

Our use of counter-stories as a theoretical lens for this work did not begin, in earnest, until data analysis had commenced. The first author had previously used Lindemann Nelson’s conceptualization of counter-stories (Lindemann Nelson, 1995, 2001) to highlight how pediatric oncology nurses re-diagnose the causes of their moral distress within institutional conditions and constraints that fractured their moral identities and inhibited them from being the “nurse [they] wanted to be” (Molinaro et al., 2023). In the current study, the child life specialists’ narratives highlighted that they told counter-stories in their daily work to continually reaffirm the value of their role to their colleagues and families receiving treatment. Noting this, and noticing that the child life specialists also told counter-stories within the narrative space of their interviews, MM brought this conceptualization of counter-stories forward to the study team, who substantiated this interpretation. From there, MM and ST interpreted the child life specialists’ narratives as instances of counter-stories against common narratives, or perceptions, of their role held by colleagues and families.

### Recruitment

After receiving ethics approval from our institutional research ethics board (HiREB #7742), we used both convenience and purposive sampling to recruit child life specialists with experience working in adult oncology and providing care for families in which an adult family member had cancer, typically in a parent or caregiver of a child or children. The professional networks of clinical team members were used to identify and recruit eligible participants. Eligible child life specialists were those who were (i) certified and practicing child life specialists in the province of Ontario; (ii) working (currently or within the past 2 years) in adult oncology; (iii) able to read, write, and communicate in English; and (iv) able to provide written informed consent. Potential participants were sent a brief email with information on the study and ST’s contact information if they were interested in participating. It is important to note that while many research approaches require large sample sizes for generalizability of findings, narrative scholars contend that the richness and depth of the stories collected have more importance than having a large quantity of stories to analyze (Connelly & Clandinin, 1990; Lieblich et al., 1998; Tuckett, 2004).

### Data Collection and Analysis

Data collection was consistent with the approach of Bertaux and Kohli (1984), two narrative scholars who recommend that story elicitation occurs over two narrative interviews. Due to public health restrictions during the pandemic, interviews were completed over Zoom or by phone. The first interview acted as the initiation of storytelling, in which the interviewer asks an open-ended question and probes in response to the stories told by the participant for the remainder of the interview (Bertaux & Kohli, 1984). In the case of our study, multiple open-ended questions were asked in the first interview, beginning by asking for stories of what it is like to be a child life specialist in adult oncology. The first interview was then transcribed verbatim and sent to each participant for review; in that email, a request for a second narrative interview was also sent.

All participants took part in a second narrative interview to elucidate additional details and stories regarding their experiences. The questions for the second interview were informed by their first interview; questions that were specific follow-up questions or probing for more detail from the first interview of each specific participant were asked first. Participants were then asked questions that were derived from analysis of all the first interviews. The second interview was also transcribed verbatim and sent to each participant for review.

Analysis methods were consistent with other narrative approaches and commenced after the transcription of the first interview (Connelly & Clandinin, 1990; Lieblich et al., 1998). Specifically, MM and ST conducted multiple close reads of the transcripts, documenting patterns to generate narrative themes (both descriptive and interpretive), taking notes about what participants were saying, how they were saying their stories, and any commonalities among the stories told. Additionally, while conducting these close readings, MM and ST made notes of any ambivalences, tensions, or contradictions that were told or suggested in the participants’ stories. After extended time reading, re-reading, and noting commonalities or differences, MM and ST generated fuller interpretations of the data, in which quotes were compiled together with notes taken about these quotes and presented to the research team. These two meetings with the research team served to query whether the interpretations presented aligned with their clinical expertise, were grounded in the data, and, in the case of this paper, made sense in relation to the conceptualization of counter-stories.

The data from all interviews were combined to generate one composite narrative, encompassing the experiences of all participants. Composite narratives allow for the generation of knowledge about a particular group of people and their experiences, by using direct quotes from all participant data to generate a single, accessible story (McElhinney & Kennedy, 2022; Wertz et al., 2011; Willis, 2019). All quotations used in this presented story are taken directly from the participants’ interviews, with only minor grammatical edits (e.g., removing repetitive words or “um”). Composite narratives are a fruitful way of exhibiting the complexities and richness of participants’ stories, while simultaneously maintaining a significant level of anonymity by combining their accounts (McElhinney & Kennedy, 2022; Wertz et al., 2011; Willis, 2019). This is particularly helpful for smaller groups of participants in identifiable roles or identity positions, such as our participants.

### Reflexivity

This study was carried out by a diverse research team, with each collaborator contributing unique skill sets. Prior to conducting the study, each collaborator took the time to reflect on their personal experiences and motivations that drove them to undertake this study. MM is a qualitative health researcher whose research explores moral distress and counter-stories of healthcare providers through a narrative lens and who took the lead on the analysis presented in this paper. MV is another qualitative health researcher who focuses on ethical complexities in various clinical contexts and substantiated the interpretations presented by MM. ST was a master’s student who brought some expertise in qualitative research and was learning narrative methodology; she was supervised by MM throughout the progression of this study and conducted the majority of the narrative interviews, as well as took part in the analysis. The broader team also included HM, a child life specialist who had prior experience working in adult oncology and brought valuable insights to the study through her personal and clinical expertise to substantiate the interpretations. DB is a research coordinator in oncology who assisted with several administrative tasks to complete this research and also offered feedback on the interpretations. DLL, a pediatric palliative care physician, and JS, a medical oncologist, both have experience working alongside child life specialists and took part in substantiating the interpretations presented with both their personal and clinical expertise.

### Findings

A total of four child life specialists who identified as women participated in our study. Their years of experience as child life specialists ranged from 1 to 14 years, and their levels of experience working in adult oncology varied between working part-time, full-time, and on an as-needed basis. Below, their stories are encompassed in the composite narrative of “Diane,” whose story was interpreted as a counter-narrative that affirmed the necessity of child life specialists in an adult oncology unit and further highlighted the importance of the role within the interprofessional healthcare team.

Diane has been a child life specialist for 8 years and has worked in adult oncology part-time for the last year and a half. When asked to story her experience as a child life specialist, her narrative began with her emphasizing that her priority is always the children of the families she is assigned to.

So as a child life specialist, when I’m supporting a family, at the forefront of my mind is the kids. I think in these situations people just don’t know what to do and go into protective mode and we really have to advocate for what the kids’ needs are. Sometimes I’m referring them to community supports or mental health organizations or just helping the family to get connected with the right people.

Part of this prioritization of the children involves her being an “interpreter” of children’s emotional and developmental needs, through advocating for the care she believes would be in the best interest of the children, communicating complex medical information to children, and helping them understand certain emotions.

Sometimes, I’m what I’ve often described as an interpreter of sorts. We take the medical information in its complexity, and we break it down into a developmentally appropriate way for each of our clients. So, if a family has several children, we may meet with each of them individually because cognitively, if the child is nine or 16, their understanding is going to be vastly different. Communication is a big part of it, as well as letting people know that emotional expression is okay. A good example with younger children is [that they think] people only cry when they’re hurt—either physically, “you punched me!” or emotionally, “you said something really sad.” Often, we’re the first to introduce them to the notion that people actually cry to release sadness, to let those emotions out which makes them feel better. So that if they see a parent crying, they have some context to understand that while it might be sad to see, it’s probably helping mom or dad feel better. The other things that we spend time doing is helping them understand that if a parent has cancer, how this is going to impact them: “What will my mom or dad look like, their physical appearance? Why is that going to happen? I don’t know if I want my mom coming to school with a bald head, my friends might make fun of me.”

By drawing attention to how building coping skills is a “big part” of the work she does with children, and further highlighting how explanations of this work are “eye-opening” for families, Diane also suggested that her work to set up children’s future abilities to regulate emotions is incredibly important to both her and the families she cares for. As she continued her story, she drew attention to how her work helps entire family units set up healthy emotional regulation and communication habits for the future.

Another big part that can happen, when there is time, is developing that emotional literacy, emotional regulation and emotional development. You’re supporting them to build coping skills for managing the really hard things and finding ways that are outlets for them and their support system. I think that’s a big part that we can address and support, when we have the time. So I think, giving some of that perspective, and often parents are like, “wow, I never really thought about that. I didn’t think about what this is going to mean to my four-year-old when he’s 20, that we involved him now.” And there can be really eye-opening moments that come from bridging each other’s perspectives.

We noted Diane’s repeated mention of “when there is time.” This phrase, to us, signaled that child life specialists are often challenged to do this work with families. We began to interpret that a lack of understanding of the role of a child life specialist, either from colleagues or from families (as described in the quote above), contributed to this lack of time to intervene.

The idea of “bridging” perspectives came up throughout Diane’s narration, which continued to emphasize the value of her role in adult oncology and the range of skills she brings to the position. In particular, she described herself as a “bridge,” connecting children with their families and families with the healthcare team to everyone together with the ultimate goal of making sure that the entire family unit is cared for. This, to us, signaled what her work is like when both colleagues and families understand the value of child life specialists in helping them navigate their understandings of the illness experience.

So, and I love this analogy—we’re also kind of like a bridge to everybody else, and it’s the most tricky, challenging bridge to build because what we find out with a family and the kids, we often will report back to their physician, to their healthcare workers, and navigate some of those issues. So, the healthcare workers might talk with us and let us know some of their concerns and then we talk with the family, so we’re kind of an in between person that that supports both the team, and the family and the kids. So, you’re almost running back and forth on the bridge, kind of like aligning with both sides and honestly, I think just trying to help kids and parents to meet on that bridge of open communication and supporting both of their needs.

Diane’s narration drew attention to how she helps both families and children make an unfamiliar experience as familiar as possible, giving families the opportunity to communicate their needs as they navigate a cancer diagnosis and what it means for the entire family unit:

I find, especially for the families who are hesitant for the kids’ involvement and are like “they’re not ready to talk about the diagnosis yet,” I’ll try to get in there and do my interventions. When I introduce myself to kids, I’ll say “my name is Diane and my job is called a child life specialist and I work here at the hospital that your mom or dad comes to. My job is to talk with families and kids just like you, who have a mum or dad that comes to this hospital because …”—if I can say they have cancer or because they’re sick or whatever the family’s comfortable with—“because we know that this can be really hard for you to have a mom or a dad that’s not that’s not well and that’s sick. My job is to help explain things to you.” And I notice these different things are being said, certain things, or it comes up in the way that they play, then I can go to the parents and tell them—“this is what I noticed, and so even though maybe you don’t think that your four-year-old understands what’s going on because no one’s told them, they know something is going on.” So that opens up opportunities for me to be able to work with the parents and say, “this is why I think we should do X, Y, Z.” Parents will be like, “we just don’t know what to say, we don’t know what to tell them,” and I’ll say “I don’t think anybody really knows any of those things until they’re put in that position and they’re forced to figure it out. That’s okay that you don’t know how to tell your child about your diagnosis, because you’ve never been in a situation like this before and nobody just wakes up and knows how to do that.”

Diane’s continued and extended narrations regarding this bridging work suggested to us that it was some of the most important work that child life specialists do. In her continuation of her “bridging” narrative, she drew attention to how this work also opens and fosters communication between all members of the family unit.

If parents have information that they want their children to know, they can use me, and what I do, to be able to communicate that. I find with families who do want involvement and want their kids involved right from the get-go, they are so involved in every aspect. I’ve worked with kids, even really little ones, who know the names of the chemo drugs. Even for those ones, questions come up with the kids that they have not been able to ask before, or they’ve not been able to articulate themselves or haven’t been given an opportunity. I find that even when the parents are encouraging open communication, they aren’t sitting down with them [their children] in a way that I or another child life specialist would to foster other opportunities for communication. So in that way, I do feel like I bridge the communication both ways, especially for kids that are really little and can’t articulate themselves or kids who might be shy. Through my work, I think I give them opportunities to express themselves in another way, and they’ve not been given that opportunity before.

It became more apparent to us that the extended time Diane took to draw attention to her abilities and scenarios of positive work with colleagues and families was done to affirm her expertise, especially as, when she continued her narration, she began to tell a multitude of stories regarding the lack of knowledge her clinical colleagues and families had about the value of child life services or specialists. It thus became more explicit to the research team that her storying of her expertise was seemingly the beginning of a counter-story to reaffirm her identity as a professional with skillsets and expertise that are valuable and necessary within adult oncology environments.

As her story continued, she explicitly countered common perceptions of her and other child life specialists by her colleagues.

There are two things that come up a fair amount that aren’t what we are or what we do. One would be our level of education. A lot of people don’t realize the level of education that child life has is not just a high school diploma or anything like that. And then, secondly, it would be that we’re not babysitters. So, there are times—and it happens more here in this adult hospital—the medical team might be having a conversation with a family and the family brings the siblings, so they might call child life and say, “can you watch the sibling while we have a conversation?” I wish people didn’t do that. I wish people knew how well educated we were and didn’t call for those things. It’s so different than the children’s hospital. I bring in teddy bears all the time as comfort items or for doing medical play or whatever, and even just walking through the halls with teddy bears—the looks that I get or comments—people always make comments. I’m bringing children’s stuff in and people are like, “what is that?,” “what is she doing?” or “those are so cute,” or “that looks so fun.” I don’t know it’s [sigh] it’s like endless, endless advocating for what the role is. I’m not here just having fun and I think that’s definitely how, a lot of times, my role is viewed. It’s tricky, I think it’s hard for people here to understand, so it’s just always explaining. I think my chart notes are so, so important for people to actually read the things that I’m doing and know the support that I’m offering, and how I’m delivering it is through play and through fun, but what I’m actually doing has a purpose and there’s a lot to it—there’s a reason.

This counter-story re-frames the narratives that Diane’s adult oncology colleagues have of her and her child life colleagues, such as those of being babysitters or child entertainers. Her narrative suggests that others within her hospital infantilize her; she perceives that others working within this hospital believe that her role is to play or have fun with the children she works with rather than understand that her work with children is often to help build emotional regulation and emotional literacy patterns came through using comfort items and engaging in medical play, such as using teddy bears. Further, her noting that she is “always just explaining” suggested that her “explaining” is the telling of a counter-story to her colleagues, to draw attention to the realities of her work and re-frame the “purpose” and necessity of her role.

She continued to counter these assumptions and drew attention to the value that the toys and teddy bears have for her work in the continuation of her story.

It’s a constant thing that I get—I think people are just curious and just not sure what I’m doing, or what’s going on kind of thing. The most common thing I get is like, “awh that’s so cute, you just get to have so much fun!” I get so many of those kinds of comments or I’ve had like three people call me Santa because I’m walking through the halls with toys. I mean I can’t expect people to understand, who just don’t know and don’t understand. [chuckles] I don’t know, I have to laugh. I feel like it’s one small step at a time of working towards people knowing the role and knowing it’s here, because people are still like “do we have a child life?” “I don’t know.” I have to brush it off. I have to. And for people who do know what I’m actually doing with the items that I have, they understand. I can only grow that one day at a time, one step a time.

Here, while sharing her counter-story in the context of a narrative interview, Diane once again draws attention to how she must continually negotiate and reaffirm her identity through sharing counter-stories at work through educating her colleagues on the importance of her role.

Sometimes, however, helping colleagues learn about the importance of her role was much harder than taking it “one step at a time.” Immediately after making the above statement, Diane narrated another negative experience with a colleague after finishing two end-of-life (parental death) cases:

There’s definitely been some days that are harder than others. I had one interaction that really, really stands out in my mind. I had two end-of-life cases on like a Thursday afternoon, so I was here late. I had done handprints and legacy work with these two families and was trying to keep everything separate. I came back to my office at the end of the day, and I had just stuff everywhere, like it was a disaster in here because I didn’t want to get things mixed up. I hadn’t cleaned anything, I was keeping everything separate, it was just like chaos. Both of these had been awful cases. Both of these were imminent deaths, I didn’t know the families before, got urgently called, like awful situations. And I was still new. I was only here for two months at this point. Anyways, a colleague walks by and was like “oh—look at you having fun with your markers in here!” Something like that. It was just such a degrading comment. And I’m very good at brushing it off now, but I think after just dealing with what I just dealt with and still being so new, I didn’t really have anybody to talk about what I just been through at that point. It felt like a kick in the gut. It was a horrible, horrible feeling. And I think it’s just an endless battle of explaining what we do and most times I can brush it off and there’s some instances, that it hits extra hard.

This counter-story draws attention to how an offhand, passing remark from a colleague not only highlighted that they have little understanding of the work the child life specialists do but further infantilized her and her role. Consequently, the “kick in the gut” and emotional toll this work took on her, in addition to this comment, made her feel unappreciated and that most of her colleagues could not understand her.

After sharing this story, she once again drew attention to how she continually has to engage in the telling of counter-stories to “educate” her colleagues on the value and necessity of her position. In doing so, her colleagues begin to view her and her colleagues as much more than “just those child people”:

Having said all of that, once I tell somebody that’s not an appropriate thing to say or call and why, they don’t do that again, or make another babysitter call. So, again it’s part of the education—of the staff—teaching them what we do. There was one time after a death where I spent the bulk of the time debriefing with the physician and organ donation person that was there and we talked about how hard that was. That conversation always starts off in many ways with another person saying, “gosh I can’t believe how hard your job is.” You know, they see us as just those child people and then when they actually witness a conversation, it’s like “holy crap. We’re not the only people to have to have really difficult conversations” and the physician on this particular weekend was crying as he was telling me that—“gosh that’s so hard.” And now that that physician and some of the other members of the team know my work, they call and ask, “when you do the death disclosure, can I come in and sit with you, so I can hear how you do it?” There was one time where the physician, the bedside nurse, and nursing student were like, “do you think we can, can we listen to how you do it?” Absolutely. And those situations made me feel very valued because they want to learn, they want to learn how to be able to do this. We’re just supporting each other. So, I just wish that people in the adult world knew about our role in general, because then they would be able to advocate for more positions and more support.

While Diane’s counter-story concludes with an example of great interprofessional collaboration between her and her colleagues, it is important to note that she must continually engage in “educating” her colleagues and “advocating” in order to reach this point. Constantly storying or expressing the vast knowledge she held about caring for children and their families was necessary—both to become more valued members of the interprofessional team and affirm that child life specialists *should be* valued members of the interprofessional team.

## Discussion

The purpose of our study was to explore and understand the experiences of child life specialists working in adult oncology. This paper presents the composite counter-story of “Diane,” consisting of interview data from the four child life specialists working in adult oncology in Ontario, Canada, who participated in our study. Diane’s narrative serves the purpose of a counter-story, attempting to repair an identity and role that has been misunderstood and mischaracterized by assumptions and systems that undermine and infantilize child life specialists and the work they do in adult oncology. Her story consistently draws attention to the skills, expertise, and value she and her work brings to an adult oncology environment, countering assumptions of her role as that of a child entertainer or a babysitter. Her narration concludes with an affirmation that, when her clinical colleagues establish a mutual understanding of the scope of her role and expertise, valuable relationships can be built and skills can be shared to ensure optimal care for adult oncology patients and families.

Diane’s storying of her role and responsibilities demonstrated an active countering of her colleagues’ lack of understanding and appreciation for her role. She describes herself as a “bridge,” connecting children with their families, and families with the healthcare team, contributing to care integration in the cancer center. This is similar to the limited body of literature on child life specialists; child life specialists have previously drawn attention to the range of skills and duties that they engage in to care for children and their families (Cole et al., 2001; Gaynard, 1985; Lookabaugh & Ballard, 2018; Wittenberg & Barnhart, 2021). The work mentioned has included assessing the interventions completed with children, helping normalize emotional expression and regulation, collaborating with other members of the healthcare team to establish goals of care for the entire family unit, and establishing communication patterns between children and their families during the time spent in the hospital in order to set them up for the future (Cole et al., 2001; Gaynard, 1985; Lookabaugh & Ballard, 2018; Wittenberg & Barnhart, 2021). In these studies, however, the outlining of their role and responsibilities was used as a comparison to highlight how child life specialists perceive their role to counter the perceptions of their clinical colleagues (Cole et al., 2001; Gaynard, 1985). Diane’s narration not only served as a counter-story but further acted as a reaffirmation that child life specialists deserve respect and appreciation for the work that they do and can do within adult oncology care environments.

Despite the significant increase in the role of child life specialists and the work they do among clinical counterparts (Wittenberg & Barnhart, 2021), our findings illustrate that there is still a persistent lack of understanding from clinical colleagues on the importance of child life specialists in adult environments. In particular, Diane’s storying of being an assumed babysitter for children is a narrative that has been reported in the literature for over three decades (Cole et al., 2001; Gaynard, 1985; Wittenberg & Barnhart, 2021). Physicians, nurses, social workers, and other healthcare professionals have previously reported that one of the primary responsibilities of child life specialists is to amuse, entertain, or babysit children when they are not involved in medical procedures (Cole et al., 2001; Gaynard, 1985; Wittenberg & Barnhart, 2021). It is important to note while this assumption can be insulting for child life specialists who are perceived in this way, the child life specialists in our study did not perceive this assumption to be one founded out of malice. Rather, our participants perceived these comments as a lack of knowledge and understanding about what is encompassed within the child life specialist role. This is consistent with previous literature examining healthcare professionals’ perceptions of child life; the perception that child life specialists were entertainers was, in part, attributed to misunderstandings surrounding play activities (Gaynard, 1985). Specifically, play activities were not understood as actual interventions that child life specialists use for clinical evaluation and assessment (Gaynard, 1985).

As Diane’s story suggested, once her clinical colleagues understood her role and responsibilities, there was a deeper respect for her work and many arising opportunities for collaboration and learning from one another. Previous literature has drawn attention to the immense value that child life specialists bring in differing clinical environments, including pediatric emergency departments (Krebel et al., 1996), pediatric inpatient units (LeBlanc et al., 2014), and pediatric radiology (Metzger et al., 2013). Cole and colleagues’ survey of 228 healthcare providers in a tertiary pediatric hospital reported that, when asked to rate the importance of their colleagues on children’s psychosocial well-being, child life specialists were ranked as most important—demonstrating the regard that their colleagues have of their ability to provide psychosocial care to pediatric patients (Cole et al., 2001). It is worth noting that in these instances, the child life specialists were employed in pediatric environments, where their presence is widely recognized and appreciated. As Diane described, in order for her to have the same level of respect and understanding from her colleagues, she must continually advocate for her role, as it is still relatively new to the adult oncology environment in which she works. While there is a paucity of literature that provides examples of successful integration of child life specialists in adult oncology, Diane’s narrative made evident what communication with families, children, and colleagues can encompass when there is respect for her role; there is opportunity for learning for all parties, and collaboration between child life specialists and their clinical colleagues can be strengthened immensely to provide better care for all members of the family.

### Strengths and Boundaries

This study makes important contributions to the body of literature examining the importance of child life specialists and their work within adult oncology environments. As there is very limited research on outcomes and experiences of child life in adult environments, our findings illustrate that child life specialists are undeservingly infantilized and mischaracterized as child entertainers. In contrast to this view, our data suggest these professionals play a crucial role in supporting not only children but also the entire family unit as they navigate a parental cancer diagnosis. Further, our use of a narrative methodology highlights how the child life specialists narrated counter-stories, situating their frustrations and these instances of infantilization in an environment that has yet to understand the importance of child life.

The findings of this research are bounded by the historical and geographical contexts in which it was conducted, as well as the concept of counter-stories used to inform the analysis. While this research is not intended to be generalizable to all child life specialists and their experiences, the findings may have less salience to child life specialists working in other clinical contexts.

## Implications for Practice

Diane’s story draws attention to the necessity of child life specialists in caring for and fostering emotional development and communication within the entire family unit. While the merits of child life specialists and the expertise they bring when caring for children experiencing illness or hospital stay are well established, their expertise has yet to be recognized in adult healthcare environments. The inclusion of child life specialists not only in adult oncology environments, but adult healthcare settings more broadly, would foster better psychosocial care for the entire family unit and offer a form of care for the children of ill parents that they would not have received otherwise. Their involvement with families can allow for the development of emotional communication and regulation skills necessary to cope with parental illness and future challenges the children may face, as well as ensure that the entire family unit is provided with developmentally appropriate psychosocial care, and an understanding of the illness that they may not have had without child life assistance.

Our findings further have implications for the integration and recognition of child life specialists in adult healthcare teams. Diane’s story drew attention to how she was the only child life specialist working in adult oncology; resultingly, much of her time was spent advocating for herself and the importance of her role within that unit. The integration of multiple child life specialists would not only allow a form of support between child life specialists to debrief on cases and share insights with one another but would further draw attention to child life specialists as valued members with skills necessary to the functioning of adult healthcare teams. Teams can enhance their integration with child life specialists by incorporating them into initial consultations with families who have children, facilitating opportunities for team members to shadow the child life specialist’s work to gauge its significance, and ensuring the presence of child life specialists during patient rounds; this in turn may simultaneously assist in promoting general awareness and understanding of the role.

## Conclusion

Our study sought to analyze the narratives of child life specialists working in adult oncology. Their stories, woven together in the composite story of Diane, drew attention to the importance of their work in adult oncology environments through the telling of a counter-story that reaffirmed her identity as a valued and necessary member of the interprofessional adult oncology team. Currently, the work of child life specialists in adult oncology environments is a relatively new initiative that can have significant positive impacts for families and their children; however, the lack of understanding from clinical colleagues of the role of child life hinders not only the development of relationships between colleagues but also the care for these families.

## Data Availability

Datasets generated for this study are not publicly available because participants did not consent to the use of their interview data beyond the research team, due to the potentially identifying nature of entire transcripts. Readers are welcome to contact Dr. Monica Molinaro for further information.

## References

[bibr1-10497323241257399] Association of Child Life Professionals . (2020). Standards of clinical practice. https://www.childlife.org/docs/default-source/aclp-official-documents/4-standards-of-clinical-practice.pdf?sfvrsn=893e8c4d_2

[bibr2-10497323241257399] Association of Child Life Professionals . (2023a). The evolution of the profession of child life in North America. https://www.childlife.org/docs/default-source/default-document-library/the-evolution-of-the-profession-of-child-life-in-north-america.pdf?sfvrsn=c048b24d_2

[bibr3-10497323241257399] Association of Child Life Professionals . (2023b). The child life profession. https://www.childlife.org/the-child-life-profession

[bibr4-10497323241257399] Baker L. Egan-Lee E. Martimianakis M. A. Reeves S. (2011). Relationships of power: Implications for interprofessional education. Journal of Interprofessional Care, 25(2), 98–104. 10.3109/13561820.2010.505350 20846045

[bibr5-10497323241257399] Bassin J. (2022). Child life at SickKids. https://www.sickkids.ca/en/care-services/support-services/child-life/#:∼:text=Certified/Child/Life/Specialists/help,reduce/fear%2C/anxiety/and/pain

[bibr6-10497323241257399] Bertaux D. Kohli M. (1984). The life story approach: A continental view. Annual Review of Sociology, 10(1), 215–237. 10.1146/annurev.soc.10.1.215

[bibr7-10497323241257399] Bissell P. Ryan K. Morecroft C. (2006). Narratives about illness and medication: A neglected theme/new methodology within pharmacy practice research. Part I: Conceptual framework. Pharmacy World and Science, 28(2), 54–60. 10.1007/s11096-006-9005-y 16649075

[bibr8-10497323241257399] Bruce J. E. McCue K. (2018). Child life in the adult ICU: Including the youngest members of the family. In *G. Netzer (Ed.),* Families in the intensive care unit (pp. 365–379). Springer.

[bibr9-10497323241257399] Bruner J. (1987). Life as narrative. Social Research, 54(1), 11–32. https://www.jstor.org/stable/40970444

[bibr10-10497323241257399] Canadian Association of Child Life Leaders . (2022). Becoming a child life specialist (CCLS). https://www.cacll.org/cls.html

[bibr11-10497323241257399] Cohen Konrad S. Fletcher S. Hood R. Patel K. (2019). Theories of power in interprofessional research–developing the field (Vol. 33, pp. 401–405). Taylor & Francis. 10.1080/13561820.2019.1669544 31566099

[bibr12-10497323241257399] Cole W. Diener M. Wright C. Gaynard L. (2001). Health care professionals’ perceptions of child life specialists. Children’s Health Care, 30(1), 1–15. 10.1207/s15326888chc3001_1

[bibr13-10497323241257399] Connelly F. M. Clandinin D. J. (1990). Stories of experience and narrative inquiry. Educational Researcher, 19(5), 2–14. 10.3102/0013189x019005002

[bibr14-10497323241257399] Crider J. Pate M. F. D. (2011). Helping children say goodbye to loved ones in adult and pediatric intensive care units: Certified child life specialist—Critical care nurse partnership. AACN Advanced Critical Care, 22(2), 109–112. 10.1097/NCI.0b013e31820810b6 21521951

[bibr15-10497323241257399] Drayton N. A. Waddups S. Walker T. (2019). Exploring distraction and the impact of a child life specialist: Perceptions from nurses in a pediatric setting. Journal for Specialists in Pediatric Nursing, 24(2), Article e12242. 10.1111/jspn.12242 30901151

[bibr16-10497323241257399] Emden C. (1998). Conducting a narrative analysis. Collegian, 5(3), 34–39. 10.1016/s1322-7696(08)60299-1 9887715

[bibr17-10497323241257399] Emerson P. Frosh S. (2004). Critical narrative analysis in psychology: A guide to practice. Springer.

[bibr18-10497323241257399] Fehlen P. (2016). Child life specialist: The chief executive officer perspective. University of Wisconsin-Stout Journal of Student Research, 15, 61–73. https://minds.wisconsin.edu/handle/1793/77636

[bibr19-10497323241257399] Gaynard L. L. (1985). Child life specialists as perceived by health care professionals (Publication No. 8523416) [Ph.D., University of Pennsylvania]. ProQuest Dissertations & Theses A&I. United States – Pennsylvania.

[bibr20-10497323241257399] Holloway D. Wallinga C. R. (1990). Burnout in child life specialists: The relation of role stress. Children’s Health Care, 19(1), 10–18. 10.1207/s15326888chc1901_2

[bibr21-10497323241257399] Krebel M. S. Clayton C. Graham C. (1996). Child life programs in the pediatric emergency department. Pediatric Emergency Care, 12(1), 13–15. 10.1097/00006565-199602000-00004 8677171

[bibr22-10497323241257399] LeBlanc C. K. Naugler K. Morrison K. Parker J. A. Chambers C. T. (2014). Parent perceptions and satisfaction with inpatient child life specialist interventions and the role of child temperament. Children’s Health Care, 43(3), 253–272. 10.1080/02739615.2013.845732

[bibr23-10497323241257399] Lieblich A. Tuval-Mashiach R. Zilber T. (1998) Narrative research: Reading, analysis, and interpretation (Vol. 47). Sage.

[bibr24-10497323241257399] Lindemann Nelson H. (1995). Resistance and insubordination. Hypatia, 10(2), 23–40. 10.1111/j.1527-2001.1995.tb01367.x

[bibr25-10497323241257399] Lindemann Nelson H. (2001). Damaged identities, narrative repair. Cornell University Press.

[bibr26-10497323241257399] Lingard L. Vanstone M. Durrant M. Fleming-Carroll B. Lowe M. Rashotte J. Sinclair L. Tallett S. (2012). Conflicting messages: Examining the dynamics of leadership on interprofessional teams. Academic Medicine, 87(12), 1762–1767. 10.1097/ACM.0b013e318271fc82 23095927

[bibr27-10497323241257399] Lookabaugh S. Ballard S. M. (2018). The scope and future direction of child life. Journal of Child and Family Studies, 27(6), 1721–1731. 10.1007/s10826-018-1031-6

[bibr29-10497323241257399] Lysecki D. Bainbridge D. Akitt T. Georgiou G. Meyer R. M. Sussman J. (2021). Feasibility of a child life specialist program for oncology patients with minor children at home: Qualitative analysis. Journal of Clinical Oncology, 39(28 Suppl.), Abstract 30. 10.1200/JCO.2020.39.28_suppl.3032822275

[bibr30-10497323241257399] McElhinney Z. Kennedy C. (2022). Enhancing the collective, protecting the personal: The valuable role of composite narratives in medical education research. Perspectives on Medical Education, 11(4), 220–227. 10.1007/s40037-022-00723-x 35951162 PMC9366824

[bibr31-10497323241257399] McMaster University Masters in Child Life & Pediatric Psychosocial Care Program . (2023). What is a child life specialist? https://childlife.healthsci.mcmaster.ca/faqs/general-faqs/

[bibr32-10497323241257399] Metzger T. Mignogna K. Reilly L. (2013). Child life specialists: Key members of the team in pediatric radiology. Journal of Radiology Nursing, 32(4), 153–159. 10.1016/j.jradnu.2013.08.001

[bibr33-10497323241257399] Mishler E. G. (1986). The analysis of interview-narratives. Praeger.

[bibr34-10497323241257399] Molinaro M. L. Polzer J. Rudman D. L. Savundranayagam M. (2023). “I can’t be the nurse I want to be”: Counter-stories of moral distress in nurses’ narratives of pediatric oncology caregiving. Social Science & Medicine, 320, Article 115677. 10.1016/j.socscimed.2023.115677 36669283

[bibr35-10497323241257399] Munn E. K. Berber C. E. Fritz J. J. (1996). Factors affecting the professional well-being of child life specialists. Children’s Health Care, 25(2), 71–91. 10.1207/s15326888chc2502_1

[bibr36-10497323241257399] Nugus P. Greenfield D. Travaglia J. Westbrook J. Braithwaite J. (2010). How and where clinicians exercise power: Interprofessional relations in health care. Social Science & Medicine, 71(5), 898–909. 10.1016/j.socscimed.2010.05.029 20609507

[bibr37-10497323241257399] Paradis E. Whitehead C. R. (2015). Louder than words: Power and conflict in interprofessional education articles, 1954–2013. Medical Education, 49(4), 399–407. 10.1111/medu.12668 25800300 PMC4405053

[bibr38-10497323241257399] Peter E. Liaschenko J. (2013). Moral distress reexamined: A feminist interpretation of nurses’ identities, relationships, and responsibilities. Journal of Bioethical Inquiry, 10(3), 337–345. 10.1007/s11673-013-9456-5 23754182

[bibr39-10497323241257399] Piazza J. Brescia A. Heering L. Jenkins J. Peters S. Dwyer-White M. Deeb G. M. (2021). Crossing the chasm to adult care settings: A journey of discovery and research. Retrieved November 24, 2021, from https://www.childlife.org/membership/aclp-bulletin/crossing-the-chasm-to-adult-care-settings-a-journey-of-discovery-and-research

[bibr40-10497323241257399] Ricks F. Faubert T. (1981). Canadian child life/non-medical programs in hospitals. Children's Health Care, 10(1), 16–19. 10.1207/s15326888chc1001_4 10252414

[bibr41-10497323241257399] Riessman C. K. (1993) Narrative analysis (Vol. 30). Sage.

[bibr42-10497323241257399] Riessman C. K. (2008). Narrative methods for the human sciences. Sage.

[bibr43-10497323241257399] Smith B. Sparkes A. C. (2006). Narrative inquiry in psychology: Exploring the tensions within. Qualitative Research in Psychology, 3(3), 169–192. 10.1191/1478088706qrp068oa

[bibr44-10497323241257399] Sutter C. Reid T. (2012). How do we talk to the children? Child life consultation to support the children of seriously ill adult inpatients. Journal of Palliative Medicine, 15(12), 1362–1368. 10.1089/jpm.2012.0019 22978620

[bibr45-10497323241257399] Taneja S. Vanstone M. Lysecki D. L. McKean H. Bainbridge D. Sussman J. Molinaro M. (2023). “There’s so much more support we could have provided”: Child life specialists’ stories of the challenges working in adult oncology. Qualitative Health Research, 14, 3253–3265. 10.1177/10497323231215950. PMC11481406 38035631

[bibr46-10497323241257399] Tuckett A. G. (2004). Qualitative research sampling: The very real complexities. Nurse Researcher, 12(1), 47–61. 10.7748/nr2004.07.12.1.47.c5930 15493214

[bibr47-10497323241257399] Vanstone M. Grierson L. (2022). Thinking about social power and hierarchy in medical education. Medical Education, 56(1), 91–97. 10.1111/medu.14659 34491582

[bibr48-10497323241257399] Wertz M. S. Nosek M. McNiesh S. Marlow E. (2011). The composite first person narrative: Texture, structure, and meaning in writing phenomenological descriptions. International Journal of Qualitative Studies on Health and Well-being, 6(2), Article 5882. 10.3402/qhw.v6i2.5882 PMC3077211 21499448

[bibr49-10497323241257399] Willis R. (2019). The use of composite narratives to present interview findings. Qualitative Research, 19(4), 471–480. 10.1177/1468794118787711

[bibr50-10497323241257399] Wittenberg B. M. Barnhart D. (2021). How are certified child life specialists perceived by healthcare professionals? A call for interprofessional collaboration. Journal of Interprofessional Care. Advance online publication. 10.1080/13561820.2020.1856053. 33472459

[bibr51-10497323241257399] Zippia . (2022). Child life specialist demographics and statistics: Number of child life specialists in the US. https://www.zippia.com/child-life-specialist-jobs/demographics/

